# Mothers with depressed mood: help-seeking from husbands and child-rearing behaviors

**DOI:** 10.1186/s12905-022-01604-5

**Published:** 2022-01-30

**Authors:** Miho Katayama, Kazuyo Kitaoka, Ritsuko Aijo

**Affiliations:** 1 Department of Nursing, Faculty of Health Sciences, Komatsu University, Mukaimotoorimachi He14-1, Komatsu City, Ishikawa Prefecture 923-0961 Japan; 2 Doctoral Course, Department of Neuroscience, Graduate School of Medical Sciences, Kanazawa University, 13-1 Takara-machi, Kanazawa City, Ishikawa Prefecture 920-8640 Japan

**Keywords:** Postpartum depression, Mothers, Parenting, Child-rearing, Child care, Grounded theory

## Abstract

**Background:**

Mothers with depressed mood tend not to seek help or support from others. Yet, there is no research providing a detailed examination of the processes that mothers with depressed mood undergo while seeking child-rearing support from their husbands. This study aimed to clarify the processes that mothers with depressed mood go through in seeking child-rearing support from their husbands and performing child-rearing duties.

**Methods:**

The participants were 10 mothers living in Japan who had given birth within the past three years and were suspected of having depression after screening using the Edinburgh Postnatal Depression Scale. Semi-structured interview data were analyzed based on the grounded theory.

**Results:**

The responses revealed that the mothers felt as though they had insufficient time for themselves, which impelled them to seek support from their husbands, consequently leading them to conclude either that child-rearing and housework are difficult or that child-rearing can be managed some way or another.

**Conclusion:**

When the husbands fully cooperated in child-rearing or demonstrated their willingness to cooperate in child-rearing, despite difficulty, the wives accepted their child-rearing support. On the other hand, if the husbands did not recognize their wives’ efforts, the wives did not accept their support, even if they had helped with child-rearing. In this way, the wives re-evaluated their relationship with the husbands based on their husbands’ attitudes toward child-rearing.

## Background

Postpartum depression is a common condition that affects about 10–30% of puerperal women [1–3]. In Japan, it is reported that 14.3% of puerperal women suffer from postpartum depression one month after giving birth [4]. Despite this, only a small percentage of mothers seek help from specialized medical institutions when they suspect that they have postpartum depression [5]. In addition, only 12.0% of mothers actually visit specialized medical institutions after being recommended to have a doctor screen them using the Edinburgh Postnatal Depression Scale (EPDS) [6], which is a screening tool for postpartum depression [7]. It is presumed that there are mothers who, despite suspecting themselves of having postpartum depression, are raising their children while in a depressed state, without receiving specialized medical care. Child-rearing provided by mothers with postpartum depression is thought to have adverse effects on the growth of their child (or children) [8] as well as on the interactions between themselves and their child(ren) [9, 10]. Thus, it is evident that postpartum depression is an important issue among the health problems that affect mothers and children, and studies must be conducted to discover what kind of support is effective in dealing with the condition.

In Japan, when women approach their due date, it is a customary practice for them to return to the homes where their parents reside, before giving birth at a maternity hospital nearby. After giving birth, women typically spend about two months at their parents’ house. For this reason, it is reported that in Japan, mothers with depressed mood select their own mothers as their first preference for supporters, followed by their husbands, and then other providers of social support [11]. However, in reality, only one in four mothers take this approach [12], and some of course do not even have the support of their mothers available for child-rearing. In some other countries, reports claim that mothers with postpartum depression first seek help from their partners [13], and support from their partners can be considered the most powerful factor in alleviating their depression [14–16]. However, no research provides a detailed examination of matters such as the processes that mothers with depressed mood undergo when they ask their husbands for support in child-rearing or the type of child-rearing support that mothers with depressed mood would find useful from their husbands. Therefore, it was necessary to take a qualitative approach to investigate the parenting of mothers with depressed moods, interviewing each individual and examining their emotions, cognitions, and behaviors.

Therefore, this study aims to identify the processes that mothers with depressed mood go through to seek child-rearing support from their husbands and perform child-rearing duties thereafter. In particular, what type of circumstances encourage mothers to seek support from their husbands and what type of conditions enable mothers to accept their husbands’ support are clarified.

## Participants, ethics, and methods

### Design

This study has a qualitative descriptive design, and the method utilizes the grounded theory approach (GTA) [17].

### Setting and participants

The study participants were 10 mothers living in Japan who were raising their children at home, after giving birth within the past three years (Table 1).

**Table 1:** Attributes of study participants

ID	Participant’s age group	Husband’s age group	No. of children	Age of children		Gave birth after returning to parents’ home	EPDS value ※1
				1st child	2nd child		
A	Early 30 s	Early 30 s	2	3 years	4 months	Yes	11
B	Late 30 s	Late 30 s	1	4 months		No	13
C	Late 30 s	Late 30 s	1	6 months		No	11
D	Late 30 s	Early 30 s	2	2 years	9 months	No	10
E	Early 40 s	Early 40 s	1	8 months		Yes	8
F	Late 30 s	Early 30 s	2	3 years	10 months	No	5
G	Late 30 s	Early 40 s	1	9 months		Yes	16
H	Early 40 s	Early 40 s	2	3 years	12 months	Yes	16
I	Early 30 s	Early 30 s	1	8 months		No	5
J	Early 30 s	Early 30 s	2	4 years	12 months	No	5

※ EPDS value at the first-month postnatal medical examination or the fourth-month postnatal home visit, the cut-off point in Japan is 9 points

EPDS: Edinburgh Postnatal Depression Scale

Since 2009, Japan has been striving to secure a healthy environment for raising infants and to prevent the isolation of families with infants by having local public health nurses conduct home visits for all families with infants aged four months [18]. In addition, under the Mother and Child Health Act [19], health examinations are also being provided to four-month-old infants free of charge at local health centers. These home visits and infant health examinations provide public health nurses the opportunity to understand the worries that mothers have about child-rearing and provide them with the support they need [20]. During these visits and examinations, public health nurses also conduct the Japanese version of the EPDS [21] on all mothers to screen for those experiencing or at risk of postpartum depression.

The target of this study is City A, which is a local hub with a population of 100,000. In City A, the aforementioned EPDS is implemented at a rate of 100%—both home visits and infant health examinations. In Japan, mothers are suspected of having postpartum depression if they obtain a score of nine or above on the EPDS [21]. In this study, the participants comprised mothers who scored nine or above on the EPDS and mothers who scored below nine but exhibited depression symptoms in clinical observations by public health nurses. Mothers with a diagnosis of postpartum depression and undergoing medical treatment were excluded.

### Data collection

Semi-structured interviews were conducted between June and December 2016 during Miho Katayama’s doctoral studies at Kanazawa University. During these interviews, the conversation centered around the following inquiries: “Looking back on your child-rearing experience since the birth, please describe the events that have occurred during child-rearing”; “Please tell us how you dealt with these events”; “Please describe the events that have happened to you while raising your child and how they made you feel.” Participants were interviewed once for about 30–60 min. In these interviews, the participants mentioned times when they sought support from their husbands. In relation to this action, 14 phenomena were identified and analyzed.

### Analysis

The GTA procedure [22] was strictly adhered to, as follows.

1. The data were read thoroughly, then a verbatim record was created and segmented into pieces of data according to the meaning of the content.
2. Properties (perspectives and viewpoints) and dimensions (positions when viewed from the properties) were extracted from each piece of segmented data, labels were given based on these properties and dimensions, and similar labels were grouped into categories.
3. To organize the several phenomena in the data, the categories were classified into situations (conditions that initiated the phenomena), actions/interactions (incidents that occurred in the situations and the handling of these incidents), and conclusions (what happened as a result).
4. The phenomena associated with the action of seeking support from the husbands were compiled and depicted in a chart that showed the relationships between the categories. Here, relationships were drawn between the categories based on their properties and dimensions. In addition, one category was selected as the **core category** at the center of the phenomena, while the others were designated as regular **categories**.
5. A storyline that uses concepts (properties and dimensions, core category, categories) to explain the chart showing the relationships between categories was created.
6. Based on a theoretical comparison (comparison of data and ideas), the following data were collected, analyzed, and integrated repeatedly. Then, a chart was created to show the integrated relationships between the categories, and a storyline was presented to explain the phenomena.

Theoretical sampling could not be implemented, as the study participants were limited to those we were introduced to, by public health nurses. To ensure the accuracy of the analysis, not only did the authors receive training on GTA, the validity of the analysis was also confirmed under the supervision of several researchers who were conducting studies using the GTA or were experts in other forms of qualitative research.

### Ethical considerations

The researchers visited the parenting classes that were being attended by candidates and gave them a direct explanation of the study’s purpose and ethical considerations. In addition, the research participants were provided a written and verbal explanation of the study’s purpose and were asked to submit a written consent form that guaranteed that their cooperation in the study would be voluntary, that they could refuse to participate at any time during or after participating in the study, and that they would not be disadvantaged in any way by refusing to participate. Furthermore, this study was approved by the Medical Ethics Committee of the Kanazawa University (approval number: 682–1). All procedures followed were in accordance with the ethical standards of the responsible committee on human experimentation (institutional and national) and with the Helsinki Declaration of 1975 (in its most recently amended version).

## Results

**Fig. 1:**
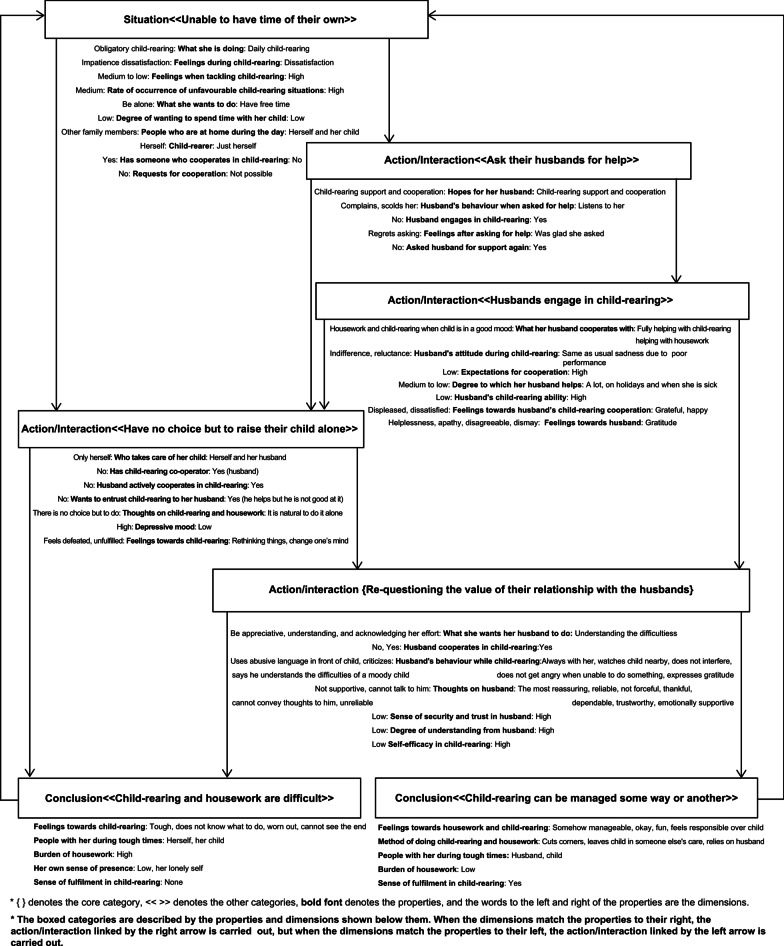
Integrated relationship concerning the core category

Several observations were made in terms of the actions exhibited by the mothers when seeking support from their husbands and practicing childcare thereafter. These phenomena, as well as the related concepts, were then extracted and organized into seven categories. The relationships between the categories were identified using properties and dimensions, and a chart was created to show the integrated relationships between the categories (Fig. 1). In Fig. 1, the boxed categories are described by the properties and dimensions shown below them. The figure displays what subsequent actions/interactions were carried out by the mothers under a certain situation where the conditions indicated by the properties and dimensions were aligned with one another. Therefore, when the dimensions matched the properties to their right, the action/interaction linked by the right arrow was carried out, but when the dimensions matched the properties to their left, the action/interaction linked by the left arrow was carried out. In addition, after writing the storyline, the core category and the other six categories are explained as follows. In the explanation, the core category is denoted with bold underlined letters, while other categories are denoted with only bold letters; the properties are denoted by “”, and the participants’ words are written in *italics*, with their participant ID included in square brackets at the end.

### Storyline: mothers with depressed mood; help-seeking from husbands and child-rearing behaviors (Fig. 1)

The mothers were raising their children each day while feeling as though they were unable to have time of their own. Consequently, the mothers underwent various processes, such as asking their husbands for help, which led them to conclude that child-rearing and housework are difficult or that child-rearing can be managed some way or another. It was found that seven processes led the mothers to conclude that child-rearing and housework are difficult, and four processes led them to conclude that child-rearing can be managed some way or another. Despite having sought support by asking their husbands for help, the mothers still concluded that child-rearing and housework are difficult. In such situation, the key point lies in re-questioning the value of their relationship with the husbands.

### The core category and the other six categories

#### Core category: re-questioning the value of their relationship with the husbands

By examining their husbands’ child-rearing behavior and involvement, the mothers re-questioned their relationship with the husbands as well as the relationships their husbands offered as parents and equal human beings. This category was the core category.

“What the mothers wanted their husbands to do” was to understand the difficulty of child-rearing. Further, if the addition of “their husbands’ child-rearing” efforts was accompanied by constant companionship, absence of interference, and words of gratitude from their husbands, the mothers felt that their husbands were reassuring, reliable, and mentally supportive, which in turn increased the degree to which “their husbands understood them”, as well as their “sense of security and trust in their husbands”. At the same time, it also increased the mothers’ “self-efficacy in child-rearing” and led them to the final conclusion that **child-rearing can be managed some way or another**. The participants expressed as follows:

Since my husband is the child’s parent, I feel that he is more reliable than my grandmother because he can manage things somehow, or rather, he is still the most reliable partner who I can entrust with child-rearing. [A]

I think my husband is the one who helps me and the one who ultimately listens to my innermost thoughts. My mood changes just by having him listen to me and talking to him. I can say anything to my husband, and he will listen. I trust my husband. [I]

What the wives wanted from their husbands were words of appreciation for their difficulties in child-rearing and acknowledgment of their daily efforts. In addition, while the wives also received their husbands’ cooperation in child-rearing, there were situations where, despite cooperating in child-rearing, the husbands would use abusive language in front of their children, criticize their wives’ child-rearing practices, insincerely express their understanding of the difficulties in child-rearing, or engage in child-rearing with a displeased look on their faces. In these situations, the wives felt that they were unable to say anything or convey their thoughts to their husbands, which consequently lowered the degree to which “their husbands understood them,” as well as their “sense of security and trust in their husbands”. At the same time, it also reduced the wives’ “self-efficacy in child-rearing” and led them to the final conclusion that **child-rearing and housework are difficult**. The participants expressed as follows:

My husband tells me that he has never seen me work hard. He says all sorts of things to me like “Ah, you’re terrible, you’re the worst,” which makes me wonder if it’s okay for me to be living my one and only life like this, and why I must be hurt so badly by the person I’m closest to. [B]

My husband says he understands child-rearing, but I do not think he actually does. I guess my husband does not support me much emotionally. I honestly wonder what it’s like for him as a husband. [G]

The other six categories are as follows:

1. **Unable to have time of their own.** These are situations where the wives were so busy with child-rearing and housework that they did not have time to even process their emotions.

Even while experiencing depressed mood, the wives still cared for their children each day, while thinking of child-rearing as an obligation. However, they were not able to perform their child-rearing duties very well. Under these circumstances, they became anxious and discontented. They did not feel very inclined to engage in child-rearing or to spend time with their children but had an increasingly strong desire to be alone. In addition, when the wives did not ask for cooperation, despite having other family members around them who could provide it, they thought that they **had no choice but to raise their children alone**. The participants expressed as follows:

The other mothers enjoy their child-rearing, but I do not. I just believe that it is my obligation to do it myself since I gave birth to my baby. Being capable of child-rearing and housework is a given to me. [A]

I feel like I am doing housework and child-rearing even when my child is sleeping. I can hardly do what I want to do. [I]

On the other hand, when things frequently went wrong during the daily child-rearing activities of the wives, they had an increased sense of failure in child-rearing. Therefore, although they still had the desire to engage in child-rearing, the wives did not feel very inclined to spend time with their child and wanted to have free time to themselves. As a result, the wives decided to **ask their husbands for help**, as they were in an environment where they were raising their child alone without any assistance. The participants expressed as follows:

My husband comes home late at seven or eight o’clock. When he comes home, he looks at our child eagerly, comforts him a bit, and then goes off to drink his own beer. It feels as though he is in charge of work, while I am in charge of the child. My husband does what he likes, and even though I think his work is tough, he is still able to do it at his own pace and comfortably relax when he gets home. However, with child-rearing, I do not have time to myself, or rather, I constantly need to think about my child first and foremost. It feels somewhat inconvenient. [C]

(2) **Have no choice but to raise their child alone.** Situations where the wives felt that they had no choice but to raise their child on their own, without relying or being able to rely on anyone.

The wives did not have a “co-operator in child-rearing” and were in a situation where they did not feel that their husbands were being cooperative. Under these circumstances, the wives lost their desire to depend on their husbands and felt a sense of resignation as well as an absence of appreciation, which compelled them to think that they had no choice but to raise their child on their own. This in turn worsened their feeling of being depressed and led them to conclude that **child-rearing and housework are difficult**. The participants expressed as follows:

My husband often told me that the situation could not be helped, that I only had myself, that I could not ask others around me for help, and that I had no choice. That is probably why I was doing things on my own. [F]

However, during such times, the mothers realized that child-rearing was not something that they were supposed to be doing on their own. Then there were situations where their husbands showed a cooperative attitude in child-rearing and provided help, despite not being very skilled at it. Consequently, this allowed wives to take the stance that child-rearing was not something that they were supposed to be doing on their own, which resulted in wives **re-questioning the value of their relationship with the husbands**. The participants expressed as follows:

There was a time when I put my child in front of me and said that I was not going to look after him anymore as he would not stop crying once he started. My husband heard me say this to myself, he had a troubled look on his face and went to sooth the child who was crying silently. [C]

(3) **Ask their husbands for help.** Situations where the wives took the action of asking their husbands for cooperation in child-rearing.

The wives took the action of asking their husbands for help. However, after doing so, their husbands rebuked the wives by telling them not to complain and did not provide cooperation. When the wives fell into this situation, they thought that they should not have said anything and never asked for help again. The participants expressed as follows:

My husband told me that he did not think that I’d complain about his cooperation in child-rearing and yelled at me (with a stunned expression) that I did not have to prepare meals (or take care of our child) if I am having a hard time. After he said that, I thought that I had failed. I gradually came to feel that it was better for me to not say anything. [B]

On the other hand, when the wives took the action of asking their husbands for help, their husbands responded by listening to the wives and helping them with child-rearing, which made them feel glad about being able to rely on their husbands. The participants expressed as follows:

I said that mum cannot be the one doing everything. I think I also said that I wanted him to do something, even when the child was not in a good mood. My husband then listened to me. [F]

(4) **Husbands engage in child-rearing.** Situations where the husbands actually engaged in child-rearing.

The husbands did housework and engaged in child-rearing only when their child was in a good mood. However, the child-rearing provided under this situation was always of low quality or characterized by poor expertise. During such instances, the husbands were either reluctant and had an indifferent attitude or were in low spirits about their poor child-rearing skills. After seeing this, the wives became dissatisfied with their husbands’ cooperation in child-rearing and felt dismayed and helpless about their husbands, which led them to think that they **have no choice but to raise their children alone**. The participants expressed as follows:

Of course, there are areas that worry me when leaving housework to my husband. In addition, there are areas that make me question whether he is doing things the right way. [A]

When our child was crying, I wanted my husband to look after her since she was crying at that moment. But it feels like my husband brings the child over to me [the mother] whenever she cries and only looks after her when she is in a good mood. [F]

The husbands would help the wives extensively with child-rearing and housework without ever changing their diligent attitude. In addition, the husbands were also highly skilled in child-rearing. During holidays and times when the wives were sick, the husbands would even help them as if it were the obvious thing to do. After experiencing this, the wives felt pleased about their husbands’ help and thanked them for it, which resulted in **re-questioning the value of their relationship with the husbands**. The participants expressed as follows:

My husband is very cooperative. He is very helpful. He puts the baby to sleep for me. He somehow does his best to put the baby to sleep and to bathe him, feed him, change his diapers, and watch over our two children when I go to the hairdresser. [H]

(5) **Child-rearing and housework are difficult.** Situations where the wives felt that child-rearing and housework were difficult and that they were unable to move around on their own.

There were wives who found child-rearing difficult. They did not know what to do and were unable to see the end to child-rearing. They felt highly burdened by their housework and were raising their children without any sense of fulfillment. In addition, the wives were carrying out child-rearing on their own while having no sense of where they were and feeling completely alone. The participants expressed as follows:

Doing so much housework and child-rearing is tough and pains me at the end of the day. [A]

It was hard not to know how much longer I had to continue this [child-rearing]. [D]

(6) **Child-rearing can be managed some way or another**. Situations where the wives had the leeway to feel that child-rearing could be managed some way or another.

The wives found housework and child-rearing to be fun and somehow manageable and were able to bear the responsibility of their children. The wives would cut corners when engaging in child-rearing duties or housework and were able to get through tasks by relying on their husbands. In addition, by having their husbands around them during difficult times, the wives could raise their children while feeling a sense of fulfillment and unity within their families. The participants expressed as follows:

Raising a child with my husband is fun, or rather, being together with my husband is the reason why I am able to continue raising our child. [A]

Generally, if I am well and if the child is well, I feel that things at home will work themselves out somehow. [F]

## Discussion

There are quantitative studies that have focused on the same purpose as our preset study. For instance, Yamada et al. [23] use a questionnaire survey to investigate the relationship between the social support received by mothers and the onset of postpartum depression. Their study depicts one causal hypothesis that the risk of developing postpartum depression increases without social support. While examining this relationship is clearly important, the GTA used in our present study has the unique feature of being able to identify various potential causal relationships. The study’s findings show that various processes occur depending on the situation, emotions, and cognitions of the mother, resulting in different consequences. In other words, we think that it depicts a phenomenon occurring in the real world.

The current study is the first to describe the processes that mothers with depressed mood go through to seek support from their husbands, who are often considered to be the people they are closest to, as well as the behavior of mothers with depressed mood when engaging in child-rearing thereafter.

In this study, these matters were examined from two perspectives that encompass the purpose of this study, that is, the circumstances that compel mothers to take the action of seeking support from their husbands and the conditions that allow mothers to accept support from their husbands.

The mothers in this study did not have time to even think about themselves, as they were busy with child-rearing duties and housework. While feeling anxious and discontented, the mothers could not find the motivation to raise their child, despite having a great sense of urgency toward child-rearing, and were incapable of giving their child love and attention, as they wanted to be alone. These attributes are symptoms of postpartum depression. Even under such circumstances, mothers with depressed mood have the tendency to not seek help from others [16, 24–27]. In the current study, the mothers did not request cooperation either, even if they had a family member who seemed capable of providing it.

Under these circumstances, there were some notable scenarios identified in this study. Mothers did boldly **ask their husbands for help**. The realization of their husbands’ freedom was thought to be the catalyst that encouraged mothers to take this action. After frequently facing situations where they could not cope in daily life while raising their child, mothers grew increasingly tired of being around their child and felt the inconvenience of living a life that revolved around their child. It is thought that while mothers were struggling alone to raise their child properly, they realized that their husbands were doing what they liked at their own pace, which made them feel dissatisfied and doubtful about how the roles in child-rearing were divided between them and their husbands. As a result, mothers asked their husbands for help.

Mothers also decided whether to accept support from their husbands depending on how their husbands responded to the request to provide help and the task of providing help. The husbands’ responses could be classified as either “refusing to help” or “complying to help”. When the mothers asked for help, the husbands refused to help them without even hearing what they had to say. Consequently, mothers regretted saying anything and stopped asking their husbands for support thereafter. For mothers with depressed mood, their husbands, who they live with, are only people from whom they take great pains to seek help [13]. Therefore, when mothers are denied support from their husbands after mustering the courage to seek support, they become socially isolated as a result [16]. In addition, raising a child in isolation has also been reported to increase the risk of abuse in child-rearing [28]. Again, when the mothers asked for help, the husbands complied to those requests. In these situations, the husbands exhibited four patterns of behavior when providing help, that is, reluctant child-rearing, extremely unskilled child-rearing, child-rearing while using abusive language and criticisms, and completely skilled child-rearing. Even when the husbands tried to provide help, they were reluctant to cooperate, had an indifferent attitude, and were doing a very poor job at helping with child-rearing and housework, which made the mothers feel dissatisfied and defeated. As a result, the mothers experienced a heightened sense of isolation [29] and ultimately chose to raise their children by themselves, as they were unwilling to accept their husbands’ cooperation in child-rearing.

However, there were husbands who, despite being very unskilled in child-rearing, continued to cooperate with their wives, without feeling discouraged or giving up; in this situation, the wives reassessed their husbands and decided to accept their support in child-rearing after seeing their diligent attitude. On the other hand, where the husbands used abusive language and pointed out flaws to their wives while assisting in child-rearing, wives gave up expecting emotional support and understanding from their husbands and were unable to accept their child-rearing cooperation as support. Therefore, it is evident that the wives were able to accept their husbands’ support in child-rearing when they provided complete and skilled cooperation in child-rearing or showed determination to cooperate, despite their poor child-rearing abilities. Conversely, when the husbands did not acknowledge their wives’ efforts, the wives were unable to accept their support, even if they helped or were ready to help with child-rearing.

Notably, the wives reassessed their husbands and re-questioned their relationship with their husbands based on their husbands’ attitude toward child-rearing. In Japan, Social roles are still highly divided based on gender. As a result, mothers spend more time raising children than fathers do [30]. Wives who become mothers spend their entire day at home, raising their children and doing more housework than before. Sometimes, mothers find that things do not work out and are tough. Therefore, they want their husbands to understand the difficulty of continuing to raise their children and doing housework when it is tough. Mothers simply want their husbands to patiently listen to how they feel [24]. During difficult times, they want to be physically and, above all else, emotionally supported. Without the support of their partners, their marital relationships will worsen [16]. It is thought that when a marital relationship shifts from being a husband-and-wife relationship between two people to a father-and-mother relationship within a family, the wives still ask whether or not they and their husbands are in a relationship with each other as partners [31]. On the other hand, husbands of mothers with postpartum depression also experience loneliness and relationship problems with their spouses [27]. If both husband and wife do not fulfill what they each require of one another, there is a considerable possibility that their married life will collapse.

In this paragraph, suggestions are made on how husbands could respond when their wives and co-parents with depressed mood symptoms seek support from them and how mothers can be encouraged to accept the support of their husbands, based on the above findings and the perspectives of health-care professionals. First, when the wives ask for cooperation in child-rearing, the husbands can support them effectively by listening to how they feel and engage in child-rearing together with them, without evaluating how they raise their child or refusing them the chance to explain themselves. It would also be more effective for the husbands to offer their cooperation in child-rearing without mothers having to ask them to begin with. Furthermore, for the mothers to accept their husbands’ support, it is important that husbands willingly listen to their grievances. Even if the husbands are unskilled in child-rearing or can only cooperate occasionally, the mothers will still want to talk to someone who is non-judgmental [257]. Hence, it is crucial that the husbands continue without giving up midway and that, above all, they appreciate their wives for enduring the hardships in child-rearing and acknowledge their efforts as well. As part of community health services, it would be effective to begin providing husbands with opportunities to receive training for child-rearing when their wives are pregnant, as it will help them to cooperate in child-rearing with ease and confidence. Additionally, to improve the situation of being **unable to have time of their own**, it would also be effective to implement a home visit childcare service, which is not yet available in Japan, to allow mothers to make time for themselves at home. This study’s findings could be used to develop questionnaires to assess husbands’ attitudes toward their wives and their level of cooperation in raising children, thereby addressing an overlooked issue in the literature. The results of research using such questionnaires could then be used to develop new treatment targets and screening tools.

This study has three major limitations. First, the sample size is small, which may make it difficult to generalize the results. Second, the participants in this study were mothers from one public health center; thus, possible influences of regional characteristics and health guidance provided cannot be eliminated. Third, because theoretical sampling was not performed, theoretical saturation may not have been reached. Focus groups could be used to validate and replicate this study’s results.

## Conclusion

While raising their children, mothers with depressed mood felt that they were **unable to have time of their own**. From there, they went through various processes before arriving at the two conclusions that **child-rearing and housework are difficult** and **child-rearing can be managed some way or another**. Ultimately, 11 processes that led to the two conclusions were observed. The mothers sought support from their husbands after realizing that their husbands were free. The mothers accepted their husbands’ child-rearing support when the husbands provided full cooperation in child-rearing or showed their willingness to cooperate without giving up, despite their poor child-rearing abilities. On the other hand, when the husbands did not acknowledge their wives’ efforts, the wives were unable to accept their support, even if they had helped with child-rearing. In addition, the wives also started **re-questioning the value of their relationship with the husbands**, based on their husbands’ attitudes toward child-rearing.

## Data Availability

The datasets generated and/or analyzed during the current study are not publicly available in order to maintain the anonymity of the participants. The data can be made available from the corresponding author on reasonable request.

## Abbreviation

EPDS: Edinburgh Postnatal Depression Scale
